# Contribution of veterinary sector to antimicrobial resistance in One Health compendium: an insight from available Indian evidence

**DOI:** 10.3389/fvets.2024.1411160

**Published:** 2024-08-27

**Authors:** Debjit Chakraborty, Falguni Debnath, Sandip Giri, Shatabdi Saha, Soume Pyne, Raja Chakraverty, Agniva Majumdar, Alok Kumar Deb, Rajesh Bhatia, Shanta Dutta

**Affiliations:** ^1^ICMR-National Institute of Cholera and Enteric Diseases, Kolkata, India; ^2^AMR Expert, Food and Agriculture Organization, New Delhi, India

**Keywords:** antimicrobial resistance, veterinary sector, antibiotics, bovine mastitis, India

## Abstract

The application of antibiotics in the poultry and veterinary sectors is very common practice in India. Owing to the seriousness of antimicrobial resistance (AMR), the present study has illustrated the overall scenario of AMR in the poultry and veterinary sectors in India through an in-depth scoping review and key informant interview (KII). In the poultry sector, most of the studies reviewed have reported resistant bacteria isolated from chicken meat, eggs, cloacal swabs, and fecal samples, and only a few have reported the presence of resistant bacteria in and around the environment of poultry farms. The major resistant bacteria that have been reported are *E. coli*, *Salmonella* spp., *S. aureus*, *Campylobacter jejuni*, and *K. pneumoniae*. These bacterial isolates exhibited resistance to various antibiotics, such as azithromycin (21.43%), tetracycline (11.30–100%), chloramphenicol (4.76–100%), erythromycin (75–83.33%), ciprofloxacin (5.7–100%), gentamicin (17–100%), amikacin (4.76%), cotrimoxazole (42.2–60%), trimethoprim (89.4%), ceftriaxone (80%), and cefotaxime (14.29–70%). Like the poultry sector, different antibiotics are also used for treating clinical and subclinical bovine mastitis, which is one of the major problems plaguing the dairy sector. Several AMR bacterial strains, such as *E. coli*, *Staphylococcus aureus*, *S. epidermidis*, and *Klebsiella pneumoniae*, have been reported by many researchers and showed resistance against tetracycline (74%), oxytetracycline (47.37%), ciprofloxacin (51%), streptomycin (57.89%), cephalosporin (100%), and trimethoprim (70%). The KIIs have revealed several reasons behind these AMR scenarios, of which the growing need for the production of food animals and their products with inadequate infrastructure and a lack of proper knowledge on farm management among the farmers are the major ones. Though several government legislations and policies have been laid down, proper implementation of these policies, strict surveillance on antibiotic application in the poultry and veterinary sectors, awareness generation among farmers, and infrastructure development can help minimize the development and transmission of AMR bacteria within and from these sectors.

## 1 Introduction

AMR is a global public health concern and is far more complex to address due to its multifactorial nature. Though the containment measures were focused on the human health sector, off late, the environmental factors contributing to the development of AMR, particularly in developing countries have been appreciated and the need for interventions in the context of One Health approach has been felt. The One Health approach toward mitigating AMR is also important to understand its complete phenomenology. Irrational antimicrobial uses in the human health sector, along with those in the veterinary and agriculture sectors, contribute heavily to the development of resistant organisms. The veterinary sector emerged as an important area of intervention in terms of AMR containment due to the increased incidence of identification of antibiotic-resistant bacteria from different specimens of sick farm animals. Usually, in developing countries, sick farm animals are treated without consulting veterinarians and as a result, they are often misdiagnosed and wrongly treated. Another pressing issue is the application/feeding of antibiotics to farm animals in sub-therapeutic doses. Moreover, not only in episodes of sickness, farm animals are been fed antibiotics as growth promoters to meet the needs of food animals in these highly populous countries. It is projected that by 2030 the global consumption of antibiotics in animal husbandry could be approximately 200,235 tons (1), and BRICS countries (Brazil, Russia, India, China, and South Africa) would experience a 99% rise in their usage during the same period (2). This shift from extensive to intensive farming in this sector involves high usage of antibiotics. Even the identification of resistant bacteria from farm environments has become common in LMICs. Frequent exposure to low dosages of antimicrobials results in favorable conditions for the emergence of antimicrobial-resistant bacteria (ARB) in animals (2). Inappropriate application dosages of antibiotics in food animals, non-adherence to prescription (3), over-the-counter (OTC) sale of antibiotics, and failure to identify drug withdrawal period in animal food (4) enhance the emergence, spread, and contamination of ARB in food animals, which can be disseminated to human either through contact with animals/food of animal origin directly or indirectly through AMR polluted environment (5).

Very recently a group of researchers conducted a strengths, weaknesses, opportunities, and threats (SWOT) Analysis on existing policies/guidelines/regulations/legislation in India (6) related to AMR containment in India and identified several opportunities in all the contributing sectors In this communication, we attempted to examine extensive empirical pieces of evidence from different parts of India to summarize knowledge on the magnitude and pattern of resistance of AMR bacteria in the livestock sector. In addition, we tried to find out better ways to cultivate animal food while minimizing the adverse impact on human health due to antibiotic-resistant pathogens through KII. We also tried to reflect on the problem of antibiotic use in animal farming as a contributor to the Global Problem of AMR from the perspective of One Health.

## 2 Methodology of the study

### 2.1 Study design

We conducted a sequentially explanatory mixed-method study.

### 2.2 Study duration

The research was carried out from April 2021 to March 2022.

### 2.3 Study population

The articles on incidents and magnitude of AMR in the poultry/dairy/piggery sector in India, the use of antimicrobials, critically important antimicrobials in animal husbandry, and the implications of those antimicrobials on the environment published during 2010–2020 were included. KIIs were carried out with stakeholders of different strata in the animal husbandry sector.

### 2.4 Search string

(((((((((((antimicrobials) OR antibiotics) AND resistance) OR livestock rearing) OR poultry farming) OR cattle farming) OR dairy farming) OR swine farming) OR food animals) AND environment) AND India).

### 2.5 Literature review

**Figure 1:**
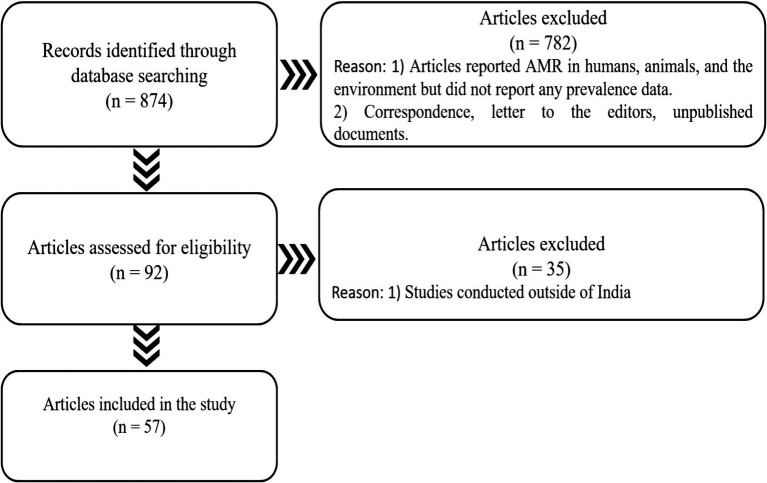
PRISMA displaying the number of papers searched, excluded, and included in the current study.

A scoping review of the literature was conducted to map the available research on AMR based on the developed criteria (Table 1), followed by the development of a KII Guide for addressing the identified gaps. The following electronic databases of published literature were searched for relevant studies: PubMed/Medline, Google Scholar, Scopus, Embase, and Index Medicus, following strict inclusion and exclusion criteria. The synthesized literature was organized by using a PRISMA flow diagram (Figure 1). We considered 57 research articles in this study.

**Table 1:** Modified PICO inclusion and exclusion criteria.

Criteria	Inclusion	Exclusion
Population	Farm animals	Not applicable
	Incidence of AMR in animals	
Outcomes	AMR in poultry	Not applicable
	AMR in dairy and pig farming	
Literature types	Indication of Antibiotic/antimicrobial or AMR should be in place	
	Articles reported the occurrence of antibiotic resistance among clinical pathogenic bacteria isolated from food animals	Articles that reported AMR in humans, animals, and the environment but that did not report any prevalence data will not be included.
	Articles reported the results of the *in-vitro* study on antimicrobial sensitivity patterns and the novel discovery of antimicrobial-resistant genes.	
	Studies conducted in India	Studies conducted outside of India
	Research articles on the development of substitutes for antimicrobials in India	Studies conducted outside of India
	Original and review articles published in peer-reviewed journals, brief reports, short communications, mini-reports, published thesis	Correspondence, letter to the editors, unpublished documents
Study period	Publications from the year 2010 to 2020	Studies published before 2010

### 2.6 Interview with stakeholders

For conducting KIIs in the animal husbandry sector, stakeholders were chosen based on their proficiency in their field and readiness to share information. A total of three KIIs (two veterinarians and one extension officer) were conducted with an interview guide, which contained open questions with probes in the following segments: existing scenario of antibiotic application on farm animals; factors influencing AMR; ways to contain AMR in animal farming in India; and best practices in India to substitute antimicrobial application on animal farming. Each interview was audio-recorded and transcribed into text data in the local language first, then back-translated to English by the separate investigator. Afterward, all transcriptions were organized systematically and coded based on the predetermined gaps of the scoping review. The distinct codes were then assembled by removing redundancy to develop the key themes.

## 3 Results

### 3.1 Antimicrobial resistance in poultry in India

**Figure 2:**
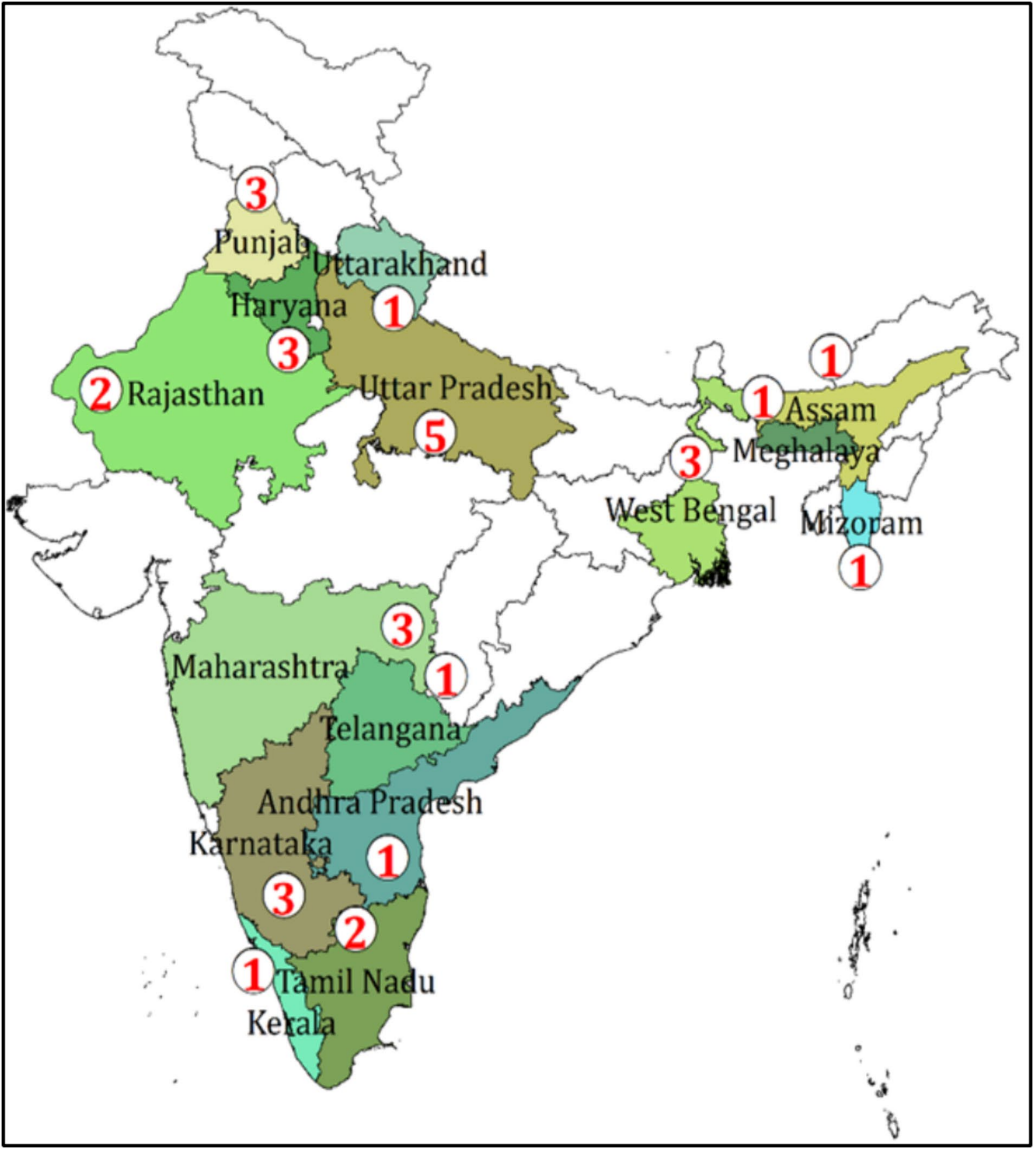
Spatial distribution of the number of studies on AMR from the poultry sector in India, 2010–2020.

Among the available original research, most of them are concerned about bacteria collected from the samples of poultry birds including meat, eggs, cloacal swabs, and fecal samples, and few of them reported the presence of resistant bacteria in the drinking water of the farm. The spatial distribution of the study shows the evidence generated from almost all regions of India (Figure 2).

**Figure 3:**
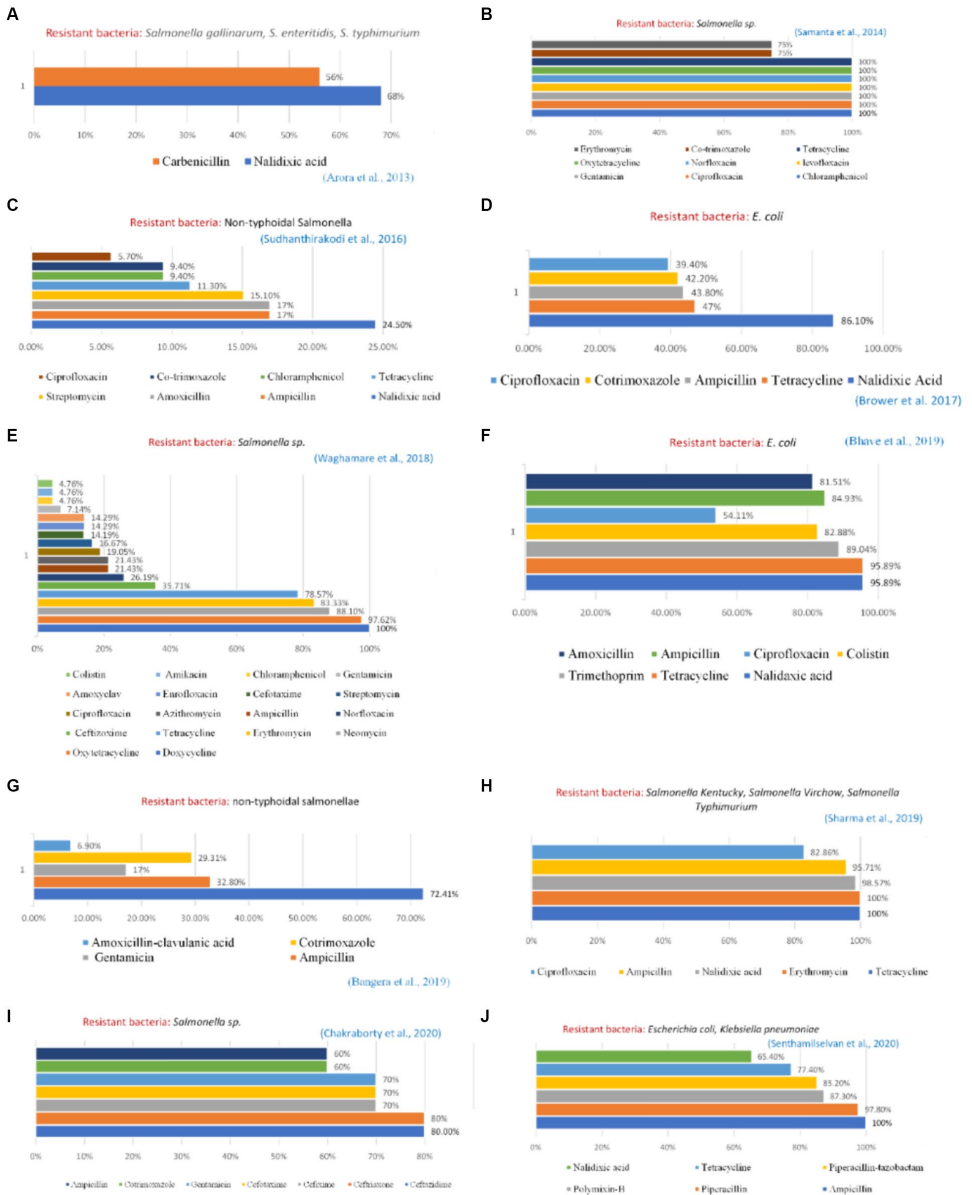
Profile of resistant bacteria from the poultry sector, India, 2010–2020. Amr instances reported from — **(A)** Haryana **(B)** West Bengal **(C)** West Bengal **(D)** Punjab **(E)** Maharashtra **(F)** Maharashtra **(G)** Karnataka **(H)** Uttrakhand and Uttar Pradesh **(I)** Mizoram **(J)** Tamil Nadu.

Reported studies identified *Salmonella* sp. from various chicken organs in Kerala which was completely resistant to the antibiotic erythromycin. Singh et al. (15) discovered the prevalence of a resistant strain of *Salmonella typhimurium* with 100% resistance to clindamycin, oxacillin, penicillin, and vancomycin in the cloacal swab, feed, water, eggs, and feces of chickens in Bareilly, Uttar Pradesh. Studies carried out in states such as Tamil Nadu (7) and Kolkata (8) generated increased evidence of bacteria from poultry samples demonstrating high resistance to broad-spectrum antibiotics such as tetracycline, nalidixic acid, ciprofloxacin, levofloxacin, ofloxacin, and sulfamethoxazole-trimethoprim (Figure 3). Several genes have also been reported which are responsible for developing resistance among the bacterial strains. In *Salmonella* sp. *tetA and tetB, bla*TEM and CTX-M genes were reported to develop resistance against tetracycline, broad-spectrum β-lactamases, and β-lactamases with extended-spectrum, respectively (9). Similarly, for *E. coli* isolates some β-lactam genes such as *bla*TEM, *bla*CTX-M, and *bla*OXA were reported which showed resistance against a wide range of commonly used antibiotics such as nalidixic acid, trimethoprim, tetracycline, colistin, and ciprofloxacin, including β-lactam antimicrobials such as amoxicillin and ampicillin (10).

### 3.2 Antimicrobial resistance (AMR) in dairy and piggery in India

**Figure 4:**
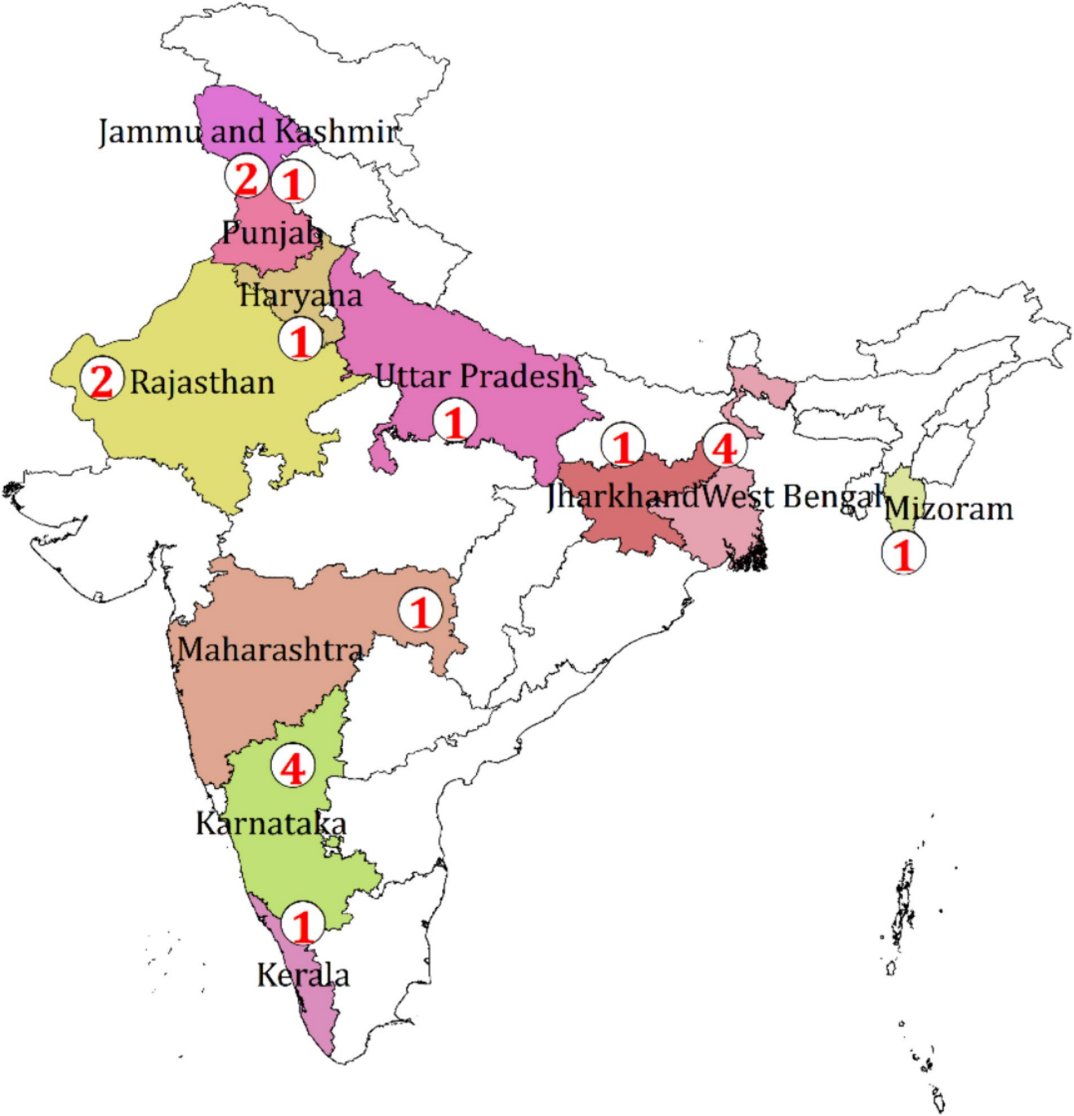
Distribution of the number of studies on AMR from the dairy sector in India, 2010–2020.

Figure 4 shows the distribution of the total number of collected original studies on AMR published in the dairy and piggery sectors in India. Mastitis has been the primary focus of most of the research on AMR in the dairy industry because of evidence of bacterial-resistant strains of mastitis in cow milk.

*Escherichia coli* resistance was reported by Thaker et al. (11) to ampicillin, streptomycin, and oxytetracycline (100, 57.89, and 47.37%, respectively) in cow milk. The presence of antimicrobial-resistant genes (ARGs) in bovine mastitis pathogens was the prime cause of such resistance. Bandyopadhyay et al. (12) in a West Bengal-based study explained intra-mammary infection of methicillin-resistant *Staphylococcus epidermidis, Staphylococcus aureus*, and ESBL-producing *Escherichia coli* in two Holstein Friesian (crossbred cows) with subclinical mastitis. West Bengal and Mizoram generated *Klebsiella pneumonia* in the milk of healthy cows having clinical and subclinical mastitis that are completely resistant to third-generation cephalosporin, 70% to sulpha/trimethoprim combination, 74% to tetracycline, and 51% to ciprofloxacin and piperacillin. In a study from West Bengal, *S. aureus* isolated from raw milk samples was found resistant to vancomycin (VRSA) (13). Besides slaughterhouse samples from Kerala, Mizoram, and West Bengal also reported increased resistant *S. aureus.* Several genes cause bovine mastitis, including β-lactamase genes (*bla*CTX-M, *bla*TEM, and *bla*SHV), plasmid-mediated quinolone resistance gene s (*qnrS* and *qnrB*), and sulfonamide resistance gene (*sul1*) (14).

### 3.3 Use of critically important antimicrobials in animal husbandry in India and their environmental implication

Critically important antimicrobials are those that are very frequently used for the treatment of both human and animal diseases, resulting in the emergence of ARGs, which eventually lose their effectiveness in treating people (16). The Critically Important Antimicrobials used for animal and human health issued by World Organisation for Animal Health (WOAH) and WHO (World Health Organization), has overlap of 47 antimicrobials. Many of the resistant bacteria predominant in animal reservoirs contaminate the environment through different pathways, can spread to humans, and pose a major threat to human health. The Centre for Science and Environment, Delhi, has made a list of critically important antimicrobials as well, administrated in animal husbandry in India (17).

### 3.4 Research conducted in India for developing alternatives to antibiotics application on food-producing animals

Various research institutes have attempted to develop antibiotic alternatives for producing animal food. Some salient findings of such research are summarized in Table 2.

**Table 2:** Studies reported some substitutes to the usage of antibiotics as growth promoters.

Author	Research Institute	Key findings
Debnath et al. (18)	College of Veterinary Science and Animal Husbandry, R.K. Nagar, Tripura R& D team, Ayurvet Ltd., Baddi, Himachal Pradesh	Assessed the effectiveness of herbal growth promoter’s product manufactured by M/s Ayurvet Ltd. in Baddi, India to antibiotic growth promoters in the. The product is made up of several herbal oils, including *Eruca sativa* (arugula), *Trigonella foenum graecum* (methi), *Zingiber officinale* (zinger), and *Allium sativum* (garlic). The efficacy of the product was assessed on 120-day-old chicks. It was observed that the chicks fed at the rate of 500 g/ton basal feed of herbal growth promoter presented much more growth than the control group fed with antibiotic growth promoter (Vetclin 112).
Patel et al. (19)	Sardarkrushinagar Dantiwada Agricultural University, Banaskantha, Gujarat	Studied the usefulness of the powder of *Emblica officinalis* (amla) fruit as a growth agent in commercial broiler chickens. It was documented that the chickens fed with amla powder significantly increased average body weight in 6 weeks. The fruit powder can be used cost-effectively for this purpose.
Jayaraman et al. (20)	Kemin Industries South Asia Private Limited, India West Bengal University of Animal and Fishery Sciences	Compared the growth-promoting efficacy of *Bacillus subtilis* PB6 in broiler chickens with that of antibiotic growth promoters.

### 3.5 Key informant interview (KII)

**Figure 5:**
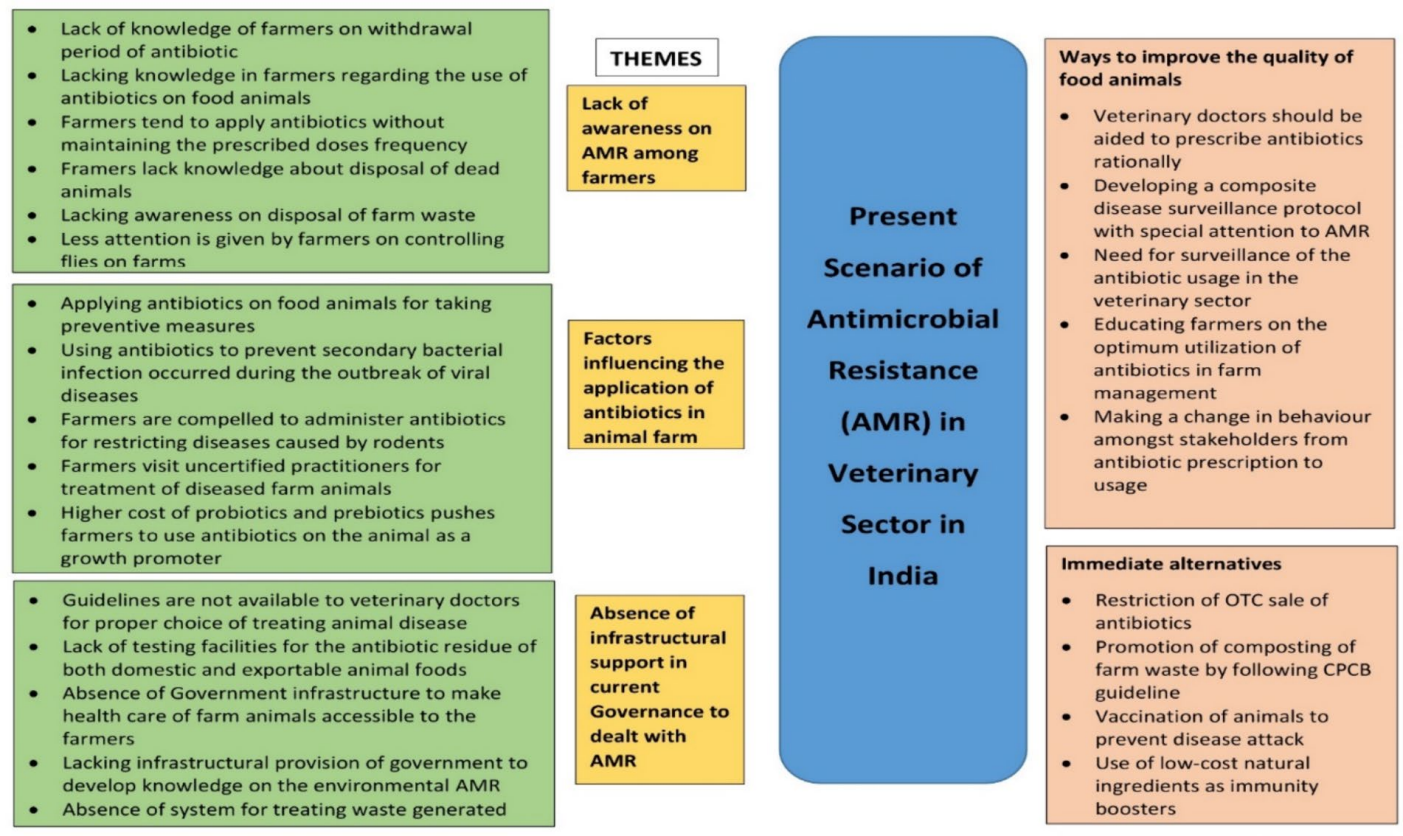
Major areas of intervention in the veterinary sector for the containment of AMR in India.

Lack of awareness of AMR among farmers, factors influencing the application of antibiotics in animal farms, and the absence of infrastructural support in current governance to deal with AMR emerged to be the major themes. These contributed immensely to the emergence and transmission of AMR bacteria in animals. Apart from having very limited awareness of the withdrawal period of antibiotics and application of them in appropriate doses farmers hardly had knowledge of farm management such as disposal of dead animals and farm waste and controlling of files. Inappropriately adopting preventive measures, restricting secondary bacterial diseases during viral outbreaks, visiting uncertified doctors to treat aliments of farm animals, preventing diseases caused by rodents, and excessive price of probiotics and prebiotics were identified as contributory factors. The stakeholders also pointed out the prerequisite of government infrastructural support including the need for guidelines to veterinarians for treating animal diseases, testing opportunities for antibiotic residue in animal food, provision by government in the development of knowledge on environmental AMR, inaccessibility of farmers to animal healthcare setup, and absence of west management in animal farms to mitigate problem such as AMR. According to the interviewees, some scopes could be created to tackle the spreading of AMR such as encouraging veterinaries to prescribe antibiotics rationally, developing a stringent disease surveillance protocol exclusively on AMR as well as the application of antibiotics in the veterinary sector, and awareness generation among farmers on the optimization of antibiotic administration in farm and making a desirable change in behavior among stakeholders toward antibiotics. To minimize the impact of AMR on the human body, some suitable alternatives were also suggested by the stakes such as checking of OTC sale of antibiotics, management of farm waste following the Central Pollution Control Board (CPCB), proper vaccination of animals, and utilization of cost-effective natural immunity boosters (Figure 5).

## 4 Discussion

The findings of the present study indicated the current scenario of AMR in the veterinary sector in India. A wide range of commonly used antibiotics such as tetracycline, erythromycin, gentamicin, chloramphenicol, ciprofloxacin, cotrimoxazole, amikacin, trimethoprim, ceftriaxone, and cefotaxime is rampantly used by the farmers in the veterinary sector. The use of antibiotic-based growth promoters in animal feed in India is a very common practice but often the farmers remain unaware of the presence of antimicrobials in the purchased feeds. Lack of proper knowledge of the farmers on antimicrobial usage is often misused by the agents and sales personnel of the different antimicrobial manufacturing companies promoting the use of antimicrobials both for prophylactic and therapeutic purposes (21). The scenario is becoming even more alarming as the critically important antimicrobials for human medicines are also being used for treating animal infections. This is the arena where AMR surveillance in the animal sector needs to be integrated with human health and antibiotic guidelines in both sectors may be made in harmony so there should not be any overlap in the use of critically important antibiotics across sectors. Despite having several guidelines and regulations on the usage of antimicrobials in animal farming in India, compliance with the same is a serious problem (5, 22–24). However, a synergy needs to be developed between different agencies such as the Bureau of Indian Standards (BIS) and the Food Standards and Safety Authority of India (FSSAI) for the reinforcement of the existing policies in controlling the unwise application of antibiotics and their residues in food animals. The practice of animal farming with less dependency on antimicrobials and shifting toward the use of India’s ethno-veterinary and traditional medicines can improve the present scenario in the long run (25, 26) if it becomes successful and economically viable, it can reduce the reliance on antimicrobials. Likewise, the ongoing research on developing herbal growth promoters in poultry can also be a future breakthrough. Investment may be made toward innovation of cost-effective alternatives to growth promoters and incentivization through subsidies may be recommended for costly alternatives at present. These initiatives will not only help to rationalize antibiotic use in animal farming but also reduce the selection pressure of antibiotics in the One Health Ecosystem Compendium.

Through KII several lacunae have been identified at the grass-root level. Farmers are randomly using antimicrobials in animal farming without proper knowledge, training, or even consulting veterinarians. Moreover, disposing of dead animals, farm manures, and litter unwisely also results in the transmission of AMR bacteria into the environment. Therefore, it is imperative to address this issue from the base level by generating awareness among the farmers about biosecurity practices such as restricted movements in the farms, quarantine and isolation of diseased or sick animals, fencing, cleaning and disinfection protocols, and diagnostic facilities (27). Animal health and AMR need to be perceived and recognized not in isolation but in the holistic and inclusive approach and as a part of global One Health initiative. The One Health approach is fully integrated into global efforts to address the problem of AMR. There are obstacles that need to be overcome including competing interests of multiple players and economic sectors including leading organizations and institutes working in animal, human, and environmental health. These actors should converge on key priorities and take calls for integrated action, find means to monitor AMR and control infections in multiple sectors, and develop cross-cutting and shared policies to govern the rational use of antimicrobials across all sectors.

## Data Availability

The raw data supporting the conclusions of this article will be made available by the authors, without undue reservation.
